# Public Interest in Distribution and Determinants of Influenza and Pneumonia Vaccination during the COVID-19 Pandemic: An Infodemiology and Cross-Sectional Study from China

**DOI:** 10.3390/vaccines9111329

**Published:** 2021-11-15

**Authors:** Liubing Gong, Xu Zhang, Zhiqiang Qu, Mark R. Francis, Kaiyi Han, Cuilin Xu, Enmao Cai, Huilin Shi, Zhiyuan Hou

**Affiliations:** 1 Chizhou Center for Disease Prevention and Control, Chizhou 247100, China; chzcpdcxf@126.com; 2 School of Public Health, NHC Key Laboratory of Health Technology Assessment, Fudan University, Shanghai 200032, China; 21211020162@m.fudan.edu.cn (X.Z.); 21211020181@m.fudan.edu.cn (Z.Q.); kaiyi.han@lshtm.ac.uk (K.H.); 3 Health Sciences Unit, Faculty of Social Sciences, Tampere University, 33014 Tampere, Finland; mark.francis@tuni.fi; 4 Department of Infectious Disease Epidemiology, London School of Hygiene & Tropical Medicine, London WC1E 7HT, UK; 5 Yuhuatai Center for Disease Prevention and Control, Nanjing 210012, China; czjmk2014@163.com; 6 Changning Center for Disease Prevention and Control, Shanghai 200051, China; caienmao@126.com; 7 School of Public Health, Key Laboratory of Public Health Safety (Ministry of Education), Fudan University, Shanghai 200032, China; 19211020057@fudan.edu.cn

**Keywords:** COVID-19, public attention, influenza vaccine, pneumonia vaccine, vaccination, determinant

## Abstract

Background: Following the COVID-19 pandemic, global interest in influenza vaccines and pneumonia vaccines has increased significantly. We aimed to examine public interest in and actual market circulation of influenza and pneumonia vaccines before and after the initial outbreak of COVID-19 and estimate the coverage and determinants of influenza and pneumonia vaccination uptake following the COVID-19 pandemic. Methods: We obtained search volume data for vaccines using the Baidu search index and collected the numbers of vaccines issued from the National Institutes for Food and Drug Control. We also conducted a cross-sectional survey among 3346 adult residents to evaluate the coverage and determinants of influenza and pneumonia vaccination uptake in the Yangtze River delta, China, from 29 January to 4 February 2021. Results: Public searches and the number of vaccines issued for the influenza vaccines and pneumonia vaccines obviously increased after the initial outbreak of COVID-19. In the total sample, 12.5% were vaccinated against influenza, and 21.5% had at least one family member vaccinated against pneumonia. A minority of participants perceived that they were highly or very highly susceptible to influenza (15.9%) and COVID-19 (6.7%). A range of socio-economic factors and perceived susceptibility to COVID-19 were associated with influenza and pneumonia vaccination uptake. Conclusions: Public interest in and issued volumes of influenza and pneumonia vaccines increased nationally following the COVID-19 pandemic. Perceptions of high susceptibility to COVID-19 were associated with the uptake of the influenza and pneumonia vaccines. Targeted interventions were needed to improve vaccination coverage.

## 1. Introduction

Influenza and pneumococcal infections place a heavy burden on communities and healthcare systems worldwide [1,2,3]. It is estimated that globally, 290,000–650,000 influenza-associated respiratory deaths occurred annually from 1999 to 2015 [2], and pneumonia was responsible for approximately 1.52 million deaths in 2015 [4] and 1.19 million deaths in 2016 [3]. Vaccination is considered as the most effective strategy for the prevention of influenza and pneumococcal infections. According to a study from the United States, vaccination prevented a significant number of influenza-related illnesses and reduced the burden of hospitalizations and death in 2017–2018, despite the low efficacy of influenza vaccines [5]. In addition, vaccination against pneumonia in adulthood reduced the burden of community-acquired pneumonia and its associated complications [6]. However, many countries face multiple challenges including vaccine shortages, a lack of public awareness, insufficient vaccine demand, and failure to integrate influenza vaccines into the national immunization program, which lead to suboptimal vaccination coverage [7,8,9,10]. For example, the influenza vaccination coverage in China was only 2% during 2004–2014 [11]. Pneumococcal vaccination coverage is also inadequate; a regional study showed that the coverage of the 23-valent pneumococcal polysaccharide vaccine (PPV23) among children and adults (above two years of age) was only 3% in Hangzhou city in 2017 [12].

In 2020, COVID-19 caused by the severe acute respiratory syndrome coronavirus 2 (SARS-CoV-2) became a pandemic worldwide. Most COVID-19 infections produce mild to moderate respiratory disease [13]. However, the constant mutations of SARS-CoV-2 could lead to recombinant variants, which will drive periodic epidemics in the years to come [14]. These spikes in COVID-19 case counts, if occurring during the months when influenza and pneumonia are prevalent [15,16], could place additional pressure on already burdened health care systems. Thus, many countries have taken measures to improve influenza and pneumonia vaccination coverage to mitigate the stress on their healthcare systems caused by the ongoing pandemic [16,17,18,19].

Several studies have investigated the public’s intention to vaccinate against seasonal influenza and pneumonia during the COVID-19 pandemic [20,21,22,23]. Some of these studies have shown that the COVID-19 pandemic might positively impact the public’s demand for seasonal influenza and pneumonia vaccinations [20,21,22]. Possible reasons for this are that influenza, pneumonia, and COVID-19 can present with similar symptoms [24,25], and beyond that, they share the same risk groups, proving detrimental to older persons and persons with chronic diseases [26,27]. Therefore, the ongoing pandemic may have contributed to a higher interest in or awareness about the influenza and pneumonia vaccines in various settings. Unfortunately, there is no research on the actual uptake of influenza and pneumonia vaccination in China since it entered the normalized epidemic prevention and control phase [28]. Therefore, timely research is needed to investigate potential links between the public interest in influenza and pneumonia vaccines and the subsequent uptake of these vaccines during the COVID-19 pandemic.

This study aimed to (1) compare public interest in influenza and pneumonia vaccines (using the Baidu search index) before and after the initial outbreak of the COVID-19 pandemic nationally, (2) estimate actual market circulation of influenza and pneumonia vaccines through data obtained from the National Institutes for Food and Drug Control (NIFDC) nationally, and (3) investigate the uptake and determinants of influenza and pneumonia vaccination following the COVID-19 pandemic through a cross-sectional survey in the Yangtze River delta.

## 2. Materials and Methods

### 2.1. Study Design

We conducted a cross-sectional survey among adults aged 18 years and above in the Yangtze River delta from 29 January to 4 February 2021. Three cities representing different levels of economic development, including Shanghai city, Nanjing city in the Jiangsu province, and Chizhou city in the Anhui province, were selected for the survey. Snowball sampling was employed to enroll the study participants. Based on the power analysis with 90% statistical power at 5% level of significance, the minimum sample size was estimated as 934 participants per city. We invited participants to complete a questionnaire through the Center for Disease Control and Prevention (CDC) and 4–8 Community Health Centers in each city. We encouraged participants who completed the survey to share a link to the questionnaire via social media platforms to invite their colleagues or friends to participate. In addition to the survey, we also assessed public interest in influenza and pneumonia vaccines using the Baidu search index [29], an internet search trends compilation and analysis tool providing daily weighted search volumes for selected keyword searches on the Baidu search engine used throughout China [30]. We overlayed these data on publicly available data on the distributed volumes of influenza vaccines (during 2018–2021) and 23-valent pneumococcal polysaccharide vaccines (2018–2020) extracted from the official website of the National Institutes for Food and Drug Control (NIFDC) [31]—the official agency responsible for the issuance of biological products, including vaccines in China. The study was approved by the Institutional Review Board of the School of Public Health, Fudan University.

### 2.2. Data Collection

We developed a web-based questionnaire to collect information on vaccination uptake and its associated factors for the cross-sectional survey. Additionally, the content validity of the questionnaire was assessed by experts in epidemiology and infectious diseases. Respondents could access the questionnaire through WeChat, a social media with 1.1 billion active users. Each WeChat account was allowed to fill in the questionnaire once to avoid data duplication, and only devices with Internet Protocol addresses in the chosen cities could successfully submit their responses to the questionnaire. The survey questionnaire was pilot tested and validated among ten respondents in a non-study community. It took approximately 3–5 min to complete the self-administered questionnaire, and respondents received electronic currency worth about CNY 5 (USD 0.77) as a gift after they completed the questionnaire. In total, 3354 participants answered the questionnaire independently and provided e-consent. Eight incomplete or less than 2 min responses were excluded from the analysis. Finally, 3346 participants were included in the present study, with an effective response rate of 99.8% (3346/3354). Using the Baidu search index, we collected daily search volumes for the keyword “influenza vaccine” for every flu season (June to March of the following year) during 2018–2021. In addition, we restricted Baidu search index data on pneumonia vaccine searches (using the keyword “pneumonia vaccine”) from January 2018 to July 2020 to avoid COVID-19 vaccine searches, which began in August 2020. We also extracted the volumes of influenza vaccines issued during 2018–2021 and 23-valent pneumococcal polysaccharide vaccines issued during 2018–2020 from the NIFDC website. The NIFDC posts updated information on its official website about the type, manufacturer, and amounts of vaccines approved for issuance. We restricted this data extraction on influenza and pneumonia vaccines issued for adults to compare the study populations.

### 2.3. Measures

For the cross-sectional survey, we collected basic socio-demographic on participants, including their gender, age, marital status, educational level, occupational, and annual household income. In addition, we used two questions to measure participants’ vaccination behavior after the COVID-19 pandemic, “Have you been vaccinated against influenza since September 2020?” and “Have you or members in your family (including the elderly and children) been vaccinated against pneumonia since 2020?”. Participants who answered “yes” to receiving an influenza vaccination were asked to select their reason for vaccination from a list of prepopulated responses.

Participants’ perceptions of their susceptibility to and the severity of influenza and COVID-19 were assessed in the questionnaire. Responses to these questions were collected on a five-point Likert scale with the following options: very high, relatively high, fair/unknown, relatively low, and very low. In addition, we outlined the response to the question measuring participant perceptions of the severity of COVID-19 using China’s Guidance on the Diagnosis and Treatment of COVID-19 [32], with the following options: severe, moderate, mild, asymptomatic, and unknown. We did not inquire about the participants’ perceptions of the susceptibility to and severity of pneumonia as it is difficult for the general population to distinguish between COVID-19 and pneumonia [14]. All the variables measuring participants’ perceptions were dichotomized for the analyses. We also assessed the participants’ familiarity with influenza vaccines and their self-rated health.

### 2.4. Statistical Analysis

We calculated frequencies and percentages to describe the study sample on unweighted data from the cross-sectional survey. We then developed univariate analyses (significance level: 0.05) using the Pearson chi-square test to examine differences in influenza (since September 2020) and pneumonia (since January 2020) vaccination uptake by the different study variables. The study variables included the participant’s socio-demographic characteristics, their perceptions about the susceptibility to and severity of COVID-19, and their self-rated health status. Participants’ perceptions of their susceptibility to and the severity of influenza and familiarity with the influenza vaccine were only investigated in the univariate analysis for the factors associated with flu vaccination uptake. All the study variables were analyzed categorically. Finally, we performed two full multivariable logistic regressions (with the “enter” method) to examine the factors associated with influenza and pneumonia vaccination uptake, and all variables analyzed in univariate analyses were added into the corresponding models for each vaccine. The model fit of logistic regression analysis was assessed using the Hosmer–Lemeshow goodness-of-fit test. The findings of the multivariate analyses are presented as adjusted odds ratios (aORs) with 95% confidence intervals (CIs). We also plotted the frequency and trends of the Baidu search index (daily weighted search volumes) for and issued volumes (monthly) of influenza and pneumonia vaccines to visually describe the public interest in and actual distribution of these vaccines before and after the pandemic. All analyses were performed using IBM SPSS Statistics for Windows, version 20.0(IBM Corp, Armonk, NY, USA).

## 3. Results

### 3.1. Baidu Search Index and the Issued Volume of Influenza Vaccines and Pneumonia Vaccines Nationally between 2018 and 2020

**Figure 1:**
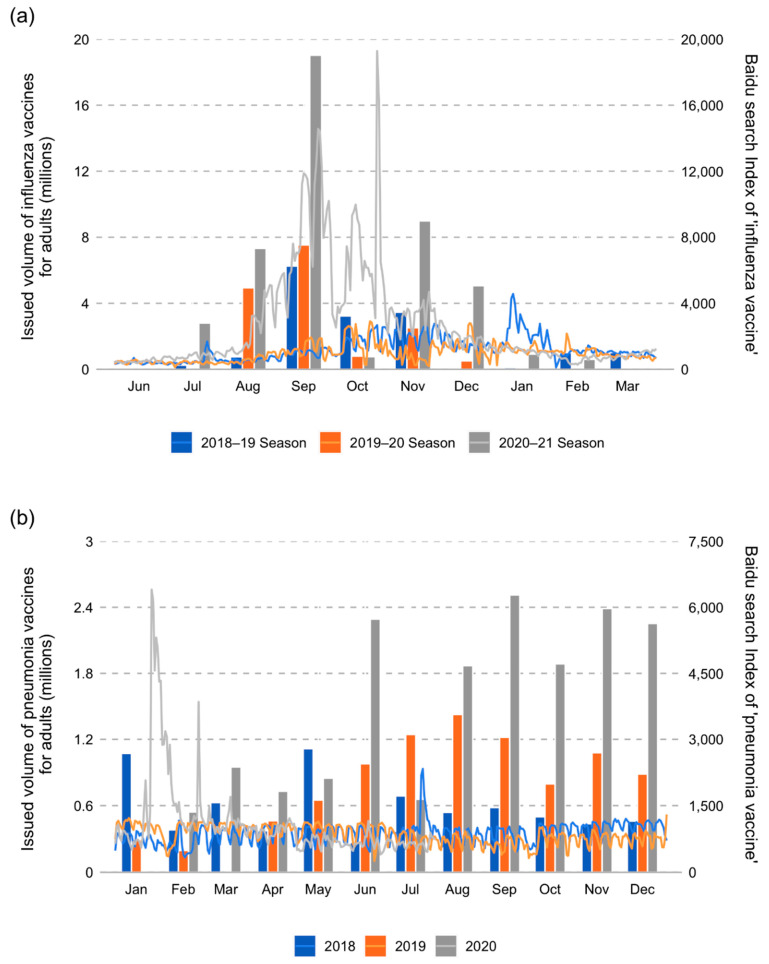
Baidu search index and issued volumes of the flu (**a**) and pneumonia (**b**) vaccines during 2018 and 2020 nationally. The trend line indicates the Baidu search index, and the columns indicate the issued volumes of the flu and pneumonia vaccines during the specified years.

The Baidu search index for the keyword ‘influenza vaccine’ suggested that public interest in the influenza vaccine was markedly higher, and the initiation of this interest was much earlier in the 2020–2021 flu season than the previous two seasons (Figure 1a). Online search volumes for influenza vaccines began to rise in August 2020, peaked in early September, and again in late October 2021. In terms of vaccine supply, the issued volume of influenza vaccines for adults in the 2020–2021 season (45.5 million) was almost three times of that in 2018–2019 (16.2 million) and 2019–2020 (16.3 million). Moreover, consistent with the Baidu search index, NIFDC began approving and issuing a larger volume of flu vaccines in July 2020, also earlier than in the previous years (Figure 1a).

For the keyword ‘pneumonia vaccine’, the Baidu search index sharply increased in late January 2020 and peaked on January 25 (6403), significantly higher than its usual search volume (around 800–900) (Figure 1b). However, it gradually decreased in early February and normalized by mid-February, except for a relatively high search volume on 25 February (3851). Thus, the public’s increasing interest in the pneumonia vaccine only lasted for about a month in 2020. The total volume of 23-valent pneumococcal polysaccharide vaccines issued for adults in 2020 (16.9 million) was around twice of that in 2018 (7.0 million) and 2019 (9.2 million) (Figure 1b).

### 3.2. Survey Sample Characteristics

A total of 3346 residents responded to the questionnaire with valid data (Table 1). Participants from Shanghai, Nanjing, and Chizhou city accounted for 32.9%, 32.6%, and 34.5%, respectively. Most respondents were female (59.9%), married (81.6%), and local registered residents (77.4%). The average age of the participants was 37.2 years, with the largest proportion aged 26–35 years (40.2%). Nearly two-thirds had obtained junior college or bachelor-level education (65.2%) and worked in government agencies or service industries (68.4%), and 56.8% had an annual household income of less than CNY 100,000 (USD 15,560). In addition, a majority of respondents considered their health status to be good (89.3%).

### 3.3. Influenza/Pneumonia Vaccination Uptake and Perceptions of Susceptibility to and Severity of Influenza and COVID-19

**Figure 2:**
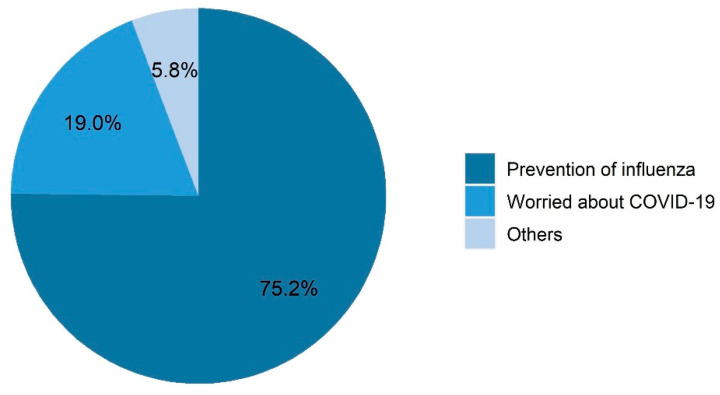
Reasons for taking the influenza vaccination during the 2020–2021 flu season in the cross-sectional survey (*n* = 415).

**Figure 3:**
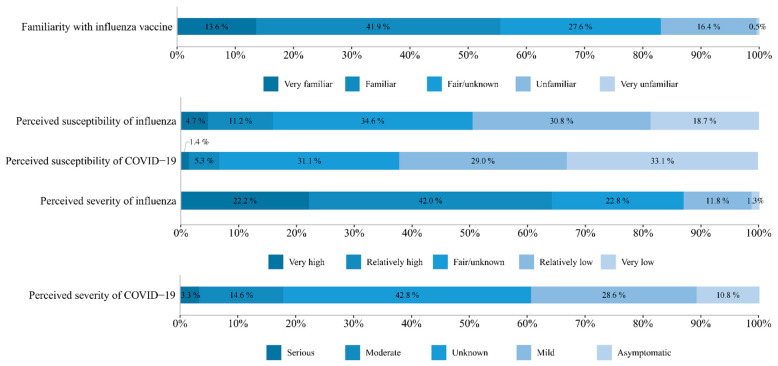
Survey respondents’ perceptions about the influenza vaccine, influenza, and COVID-19 (*n* = 3346).

About one-eighth of the respondents (12.5%) were vaccinated against influenza in the 2020–2021 flu season until the beginning of February 2021 (Table 1), and nearly one-fifth (19.0%) of these vaccinated respondents reported being vaccinated because of COVID-19 (Figure 2). In addition, 21.5% of families had at least one member vaccinated against pneumonia from 2020 until early February 2021 (Table 1). More than half of respondents considered themselves very familiar (13.6%) or relatively familiar (41.9%) with the influenza vaccine (Figure 3). Only 15.9% of the respondents considered themselves highly or very highly susceptible to influenza infections. However, more than half of the respondents considered that flu infections could be very (22.2%) or relatively highly (42.0%) serious. In addition, 6.8% considered that they were highly or very highly susceptible to COVID-19 infection, and 17.8% considered that they would be moderately or severely symptomatic if infected with COVID-19.

### 3.4. Factors Associated with Uptake of Influenza/Pneumonia Vaccination

Table 1 presents the respondents’ vaccination statuses (flu/pneumonia) by their socio-demographic and other characteristics, and the results of univariable analysis. When *p* < 0.05, the vaccination coverage of residents was significantly different between groups with different characteristics. For example, the *p* value from the Chi-square test between influenza vaccination and “familiarity with flu vaccine” was “*p* < 0.001”, which means a significantly higher proportion of participants familiar with the flu vaccine (17.8%) were vaccinated against influenza than those unfamiliar (5.8%) in the 2020–2021 flu season (until early February). It was suggested that influenza vaccination uptake in the 2020–2021 flu season (until early February) was positively associated with familiarity with flu vaccine. Such positively associated factors also included living in Nanjing or Shanghai city (*p* = 0.025), having higher education (bachelor’s degree or above) (*p* < 0.001), working in government agencies or service industries (*p* < 0.001), the highest (>CNY 200,000) or lowest (<CNY 20,000) annual household income (*p* = 0.013), and perceiving high susceptibility (*p* < 0.001) to and severity (*p* = 0.014) of influenza and high susceptibility (*p* < 0.001) to and severity (*p* = 0.033) of COVID-19. Univariable analyses also showed that the reported uptake of pneumonia vaccine was positively associated with younger age (*p* < 0.001), being married (*p* < 0.001), living in Chizhou city (*p* < 0.001), and having better self-rated health (*p* = 0.001).

Table 2 presents the results of the multivariable logistic regression conducted to assess the participant characteristics associated with influenza (2020–early 2021) and pneumonia (2020–2021) vaccination uptake. In the multivariable analysis (adjusting for participant demographics and perceptions of susceptibility to and severity of influenza and COVID-19) for flu vaccination uptake, we found that younger participants (18–25 years old) had a significantly higher likelihood (aOR: 2.32, 95%CI: 1.38–3.88) of receiving influenza vaccination. In addition, participants who were divorced or widowed (aOR: 2.06, 95%CI: 1.00–4.23), those living in Shanghai city (aOR: 1.38, 95%CI: 1.00–1.90), those familiar with the influenza vaccine (aOR: 3.02, 95%CI: 2.33–3.91), and those with a high perceived susceptibility to COVID-19 (aOR: 2.03, 95%CI: 1.42–2.89) were also significantly more likely to have received an influenza vaccination than their counterparts. However, participants who worked in the service industry (aOR: 0.58, 95%CI: 0.44–0.77), in the manufacturing industry or agriculture (aOR:0.47, 95%CI: 0.31–0.72), had an annual household income of 50–100,000 (aOR: 0.62, 95%CI: 0.42–0.89) were less likely to be vaccinated against influenza than their counterparts.

Younger participants (18–25 and 26–35 years old) were also observed to have a higher likelihood of pneumonia vaccination for their family members (Table 2). Similarly, participants who were married (aOR: 3.42, 95%CI: 2.41–4.85), or divorced or widowed (aOR:2.11, 95%CI: 1.02–4.39), those with better self-rated health (aOR: 1.49, 95%CI: 1.09–2.03) and higher perceived susceptibility to COVID-19 (aOR: 1.42, 95%CI: 1.01–1.98) had an increased likelihood of receiving pneumonia vaccination for their family members. However, participants living in Nanjing city (aOR: 0.65, 95%CI: 0.52–0.82), those working in the service industry (aOR: 0.78, 95%CI: 0.63–0.98) and the manufacturing industry or agriculture (aOR: 0.70, 95%CI: 0.52–0.96) were less likely to have their family members receive a vaccination against pneumonia than their counterparts.

## 4. Discussion

Our study investigated public interest in and distribution of influenza and pneumonia vaccines nationally and assessed the coverage and determinants of these vaccines among residents in the Yangtze River delta following the COVID-19 pandemic. After the COVID-19 pandemic, we found that public interest in and the issued volumes of the influenza vaccine had increased and peaked earlier than the respective flu seasons nationally. The cross-sectional survey revealed that influenza vaccination coverage among adults was low, with 12.5% of participants reporting vaccination during 2020 and early 2021 (until February 2021). In addition, a minority of participants perceived that they were highly or very highly susceptible to influenza (15.9%) and COVID-19 (6.7%). While more than half (64.2%) of the participants perceived a high severity of influenza, only 17.9% reported that COVID-19 infections could be severe. Furthermore, while participant characteristics such as younger age, being widowed or divorced, living in Shanghai, familiarity with the flu vaccines, and high perceived susceptibility to COVID-19 was positively associated with flu vaccination uptake, certain occupations (service or agricultural industry), and reporting a higher annual household income (CNY 50–100,000) were negatively associated with flu vaccination uptake during 2020 and early 2021. In addition, public interest in the pneumonia vaccine had a significant but short-term increase immediately following the COVID-19 pandemic. The total issued volume of PPV23 in 2020 was also higher than that in 2018 and 2019. Pneumonia vaccination coverage among the surveyed participants or their family members was 21.5%, and younger age, being married, divorced, or widowed, and high perceived susceptibility to COVID-19 were positively associated with pneumonia vaccination uptake for themselves or their family members. However, respondents living in Nanjing city and those working in the service industry or agricultural industry had a lower likelihood of pneumonia vaccination for themselves or their family members.

Public interest in and issued volumes of the influenza vaccine and pneumonia vaccine increased nationally during the COVID-19 pandemic. In 2018–2019, we found that the search volume of influenza vaccines increased gradually from September. However, after the initial outbreak of COVID-19, public interest in the influenza vaccine peaked earlier than before. As a result, there was a sharp increase in search volume for influenza vaccines from August to November 2020, and there was also a significant but short-term increase in the search volumes for pneumonia vaccines from late January to early February 2020, consistent with the findings of Paguio J A et al. [33]. The daily search volumes for the influenza vaccine peaked on September 23 and October 26, which represented the highest volume of online searches for influenza vaccines during the study period. The daily search volumes for pneumonia vaccines peaked on January 25, 2020. Furthermore, data from NIFDC on issued volumes of the influenza vaccine for adults were advanced to July in 2020, and the final number of vaccines issued in 2020 was nearly three times the number issued in the previous year, and the issuance volume of PPV23 in 2020 was about twice that in 2019. Similarly, in Japan, the PPV23 issued in the first three quarters of 2020 (156.6 thousand) was 2.5 times that in 2019 (62.3 thousand), indicating an increased demand for pneumonia vaccination [34]. Although the Chinese government has increased the supply of influenza vaccines, it still has not met the residents’ demand for influenza vaccination. According to public reports [35], due to the sudden surge in domestic demand for influenza vaccines in 2020, there were shortages of flu vaccines in many Chinese cities. The public interest in the flu vaccine seemed to be correlated with the increased demand following the COVID-19 pandemic. Further research could better use secondary data sources, such as the Baidu search index and the NIFDC repository, to monitor public interest and optimize vaccine supply to meet increasing demand. The increase in demand for flu vaccines after the COVID-19 pandemic provides an opportunity to bolster public efforts to promote influenza and pneumonia vaccination across the country.

Our results reveal that influenza vaccination coverage among adults in Shanghai, Nanjing, and Chizhou cities was 12.5% during the 2020–early 2021 flu season. However, since the 2020–2021 flu season was not yet completed at the time of this survey, it is expected that final vaccination rates for the completed flu season would be higher. Despite being low, influenza vaccination coverage in our study is higher than in previous studies [36]. In the study of Chu et al. [37] conducted among American adults, the flu vaccination coverage in 2020–2021 flu season was slightly higher than the coverage reported by CDC in 2019–2020 flu season, which is similar with our results. In Australia, the flu vaccine uptake of adults aged ≥ 65 between March and August 2020 is also higher than in 2019 [38]. We found that respondents who perceived themselves as highly susceptible to COVID-19 were two times more likely receive an influenza vaccination, and one-fifth (19%) of participants reported being “worried about COVID-19” as their main reason for being vaccinated. This finding is in some agreement with other international studies. Domnich et al. [20] found that about 20.4% of the Italian adults would not receive the 2020/21 flu vaccine if no COVID-19 pandemic occurred, and Marcus, R.E [39] found that 12.5% of adults in a rural American community changed their flu vaccination plan due to COVID-19. Perceptions of high susceptibility to COVID-19 were also positively associated with pneumonia vaccination uptake in our survey. The increased demand for the flu and pneumonia vaccines may be due to perceived similarities between influenza or pneumonia and COVID-19 infections, the severity of co-infections, or beliefs in the protective effects these vaccines offer against COVID-19 [16,40,41,42,43]. Furthermore, previous research has shown that health professionals may influence their patient’s awareness about the influenza vaccine during COVID-19 consultations, which may serve as a cue to action [44]. While the COVID-19 pandemic has subsided in China since May 2020 [28], SARS-CoV-2 virus infections may peak every fall/winter, along with influenza. Therefore, measures that focus on the ongoing pandemic and on sustaining or improving routine immunization must be introduced to ensure the uptake of influenza, pneumonia, and other vaccines.

Influenza vaccination coverage in our study was higher among younger respondents, those with high perceived susceptibility to influenza and familiarity with the flu vaccine, and government agency employment. Similar associations between younger age and government agency employment were also positively associated with pneumonia vaccination uptake, along with marital status and good self-rated health. Cognitive differences may explain the differences in influenza and pneumonia vaccination uptake by age group [45]. Young adults may be more concerned about their health and have greater access to health information; consequently, their understanding and acceptance of vaccines may be higher. The associations between occupation and vaccination uptake may be due to the relatively high level of education and health literacy of people working in a government agency. The associations between familiarity with the flu vaccine and perceived high susceptibility to influenza and influenza vaccination uptake were consistent with previous research [46,47,48,49]. Furthermore, the observed association between self-rated health and pneumonia vaccination uptake is consistent with a regional study from China [50], which found a higher willingness to be vaccinated among the elderly with good self-reported health. Taken together, these findings can help identify the population to target for concerted public health efforts to increase routine vaccination coverage in urban China.

We also found significant regional differences in influenza vaccination coverage after controlling respondents’ socio-demographic characteristics and perceptions. Influenza vaccination coverage was highest in Nanjing, followed by Shanghai and Chizhou city. These differences may be due to the disparate pricing or availability of adults’ influenza vaccines in these cities. For example, influenza vaccine-related information and appointments for vaccinations can be accessed on public platforms such as WeChat in Nanjing and Shanghai, which may increase accessibility to influenza vaccination services. In addition, as the influenza vaccine is self-paid in China, the vaccine’s price, supply, and quality of vaccination services may also affect residents’ acceptance of and willingness to be vaccinated [51,52,53]. However, interestingly, we found that pneumonia vaccination coverage from 2020–early 2021 was higher in Chizhou (27.5%), compared with that in Nanjing (17.1%) or Shanghai (19.6%). Further research is needed to explain these disparities in influenza and pneumonia vaccination uptake by region.

Our study examined public interest in and issued volumes of influenza and pneumonia vaccines nationally during the COVID-19 pandemic and assessed the coverage and determinants of these vaccines in the Yangtze River delta. These findings can help guide public health efforts to improve influenza and pneumonia vaccination coverage in the Yangtze River delta. However, our study has certain limitations. First, our study sample may have a degree of selection bias due to the snowball sampling and use of an online questionnaire. Consequently, younger participants may have been overrepresented as they are more active on the Internet. Additionally, our dissemination of questionnaires using the local CDC and through vaccination clinics may have led to the recruitment of respondents with high health literacy. Therefore, our survey may not perfectly represent the opinions of all residents in the Yangtze River delta. Second, participants may report higher protective behaviors, such as vaccinations, due to a social-desirability bias, especially during the ongoing pandemic. Third, we did not investigate the impact of access to vaccination services on vaccination behaviors, including vaccine supply and accessibility. Lastly, the survey ended in January 2021 and did not cover the completed 2020–2021 influenza season.

## 5. Conclusions

Our survey showed that public interest in and issued volumes of influenza and pneumonia vaccines significantly increased nationally following the COVID-19 pandemic. We also found that influenza vaccination coverage among respondents from three regions of the Yangtze River delta was higher than in previous reports before the COVID-19 pandemic but still suboptimal. In addition, participants reported a low perceived susceptibility to influenza and COVID-19, and perceptions of high susceptibility to COVID-19 were associated with the uptake of the flu and pneumonia vaccines. Other socio-demographic characteristics such as age, residence, employment, and marital status were also associated with vaccine uptake. These findings point to the potential for targeted interventions to improve vaccination coverage, especially in the backdrop of the COVID-19 pandemic. More in-depth research is needed to utilize online search data, such as the Baidu search index, to monitor public attention and optimize vaccine supply to meet residents’ increasing demands. Moreover, measures that can deal with the ongoing pandemic and maintain routine vaccination services should be introduced and maintained to ensure the regular delivery of influenza, pneumonia, and other vaccines.

## Figures and Tables

**Table 1:** Univariable analysis of the respondents’ characteristics and their flu/pneumonia vaccination status. n (%).

Characteristics	Total	Received a Flu Vaccination during 2020–Early 2021 Season (Until Early February 2021)		Self or Family Member Received a Pneumonia Vaccination during 2020–Early 2021	
		Yes	No	Yes	No
Total	3346	417 (12.5)	2929 (87.5)	720 (21.5)	2626 (78.5)
Gender		*p* = 0.138		*p* = 0.211	
Male	1341 (40.1)	181 (13.5)	1160 (86.5)	274 (20.4)	1067 (79.6)
Female	2005 (59.9)	236 (11.8)	1768 (88.2)	446 (22.2)	1559(77.8)
Age		*p* = 0.076		*p* < 0.001	
18–25	341 (10.2)	50 (14.7)	291 (85.3)	71 (20.8)	270 (79.2)
26–35	1346 (40.2)	147 (10.9)	1199 (89.1)	341 (25.3)	1005 (74.7)
36–45	927 (27.7)	131 (14.1)	796 (85.9)	190 (20.5)	737 (79.5)
≥46	732 (21.9)	89 (12.2)	643 (87.8)	118 (16.1)	614 (83.9)
Marital status		*p* = 0.495		*p* < 0.001	
Single	532 (15.9)	65 (12.2)	467 (87.8)	62 (11.7)	470 (88.3)
Married	2730 (81.6)	338 (12.4)	2392 (87.6)	647 (23.7)	2083 (76.3)
Divorced/widow	84 (2.5)	14 (16.7)	70(83.3)	11 (13.1)	73 (86.9)
Living city		*p* = 0.025		*p* < 0.001	
Chizhou	1153 (34.5)	119 (10.3)	1034 (89.7)	317 (27.5)	836 (72.5)
Nanjing	1091 (32.6)	149 (13.7)	942 (86.3)	187 (17.1)	904 (82.9)
Shanghai	1102 (32.9)	149 (13.5)	953 (86.5)	216 (19.6)	886 (80.4)
Local registered residents		*p* = 0.366		*p* = 0.115	
Yes	2590 (77.4)	330 (12.7)	2260 (87.3)	573 (22.1)	2017 (77.9)
No	756 (22.6)	87 (11.5)	669 (88.5)	147 (19.4)	609 (80.6)
Education		*p* < 0.001		*p* = 0.127	
Middle school or below	574 (17.1)	59 (10.3)	515 (89.7)	122 (21.3)	452 (78.7)
High school or technical secondary school	593 (17.7)	63 (10.6)	530 (89.4)	148 (25.0)	445 (75.0)
Junior college	735 (22.0)	75 (10.2)	660 (89.8)	145 (19.7)	590 (80.3)
Bachelor’s degree or above	1444 (43.2)	220 (15.2)	1224 (84.4)	305 (21.1)	1139 (78.9)
Occupation		*p* < 0.001		*p* = 0.105	
Government agency	1179 (35.2)	219 (18.6)	960 (81.4)	277 (23.5)	902 (76.5)
Service industry	1111 (33.2)	121 (10.9)	990 (89.1)	216 (19.4)	895 (80.6)
Manufacturing industry or agriculture	376 (11.3)	31 (8.2)	345 (91.8)	76 (20.2)	300 (79.8)
Others (students, unemployed et al.)	680 (20.3)	46 (6.8)	634 (93.2)	151 (22.2)	529 (77.8)
Annual household income (CNY)		*p* = 0.013		*p* = 0.592	
<20,000	418 (12.5)	59 (14.1)	359 (85.9)	90 (21.5)	328 (78.5)
20–50,000	515 (15.4)	59 (11.5)	456 (88.5)	112 (21.7)	403 (78.3)
50–100,000	966 (28.9)	96 (9.9)	870 (90.1)	219 (22.7)	747 (77.3)
100–200,000	857 (25.6)	111 (13.0)	746 (87.0)	168 (19.6)	689 (80.4)
>200,000	590 (17.6)	92 (15.6)	498 (84.4)	131 (22.2)	459 (77.8)
Self-rated health		*p* = 0.342		*p* = 0.001	
Good	2988 (89.3)	378 (12.7)	2610 (87.3)	667 (22.3)	2321 (77.7)
Fair or poor	358 (10.7)	39 (10.9)	319 (89.1)	53 (14.8)	305 (85.2)
Familiarity with flu vaccine		*p* < 0.001		-	-
Familiar	1856 (55.5)	330 (17.8)	1526 (82.2)		
Unfamiliar	1490 (44.5)	87 (5.8)	1403 (94.2)		
Perceived susceptibility of flu		*p* < 0.001			
High	530 (15.8)	100 (18.9)	430 (81.1)		
Low	2816 (84.2)	317 (11.3)	2499 (88.7)		
Perceived severity of flu		*p* = 0.014			
Severe	2147 (64.2)	290 (13.5)	1857 (86.5)		
Slight	1199 (35.8)	127 (10.6)	1072 (89.4)		
Perceived susceptibility of COVID-19		*p* < 0.001		*p* = 0.217	
High	226 (6.8)	63 (27.9)	163 (72.1)	56 (24.8)	170 (75.2)
Low	3120 (93.2)	354 (11.3)	2766 (88.7)	664 (21.3)	2456 (78.7)
Perceived severity of COVID-19		*p* = 0.033		*p* = 0.478	
Severe	597 (17.8)	90 (15.1)	507(84.9)	122 (20.4)	475 (79.6)
Slight	2749 (82.2)	327 (11.9)	2422(88.1)	598 (21.8)	2151 (78.3)

Note: *p*-value represents the result of each univariable analysis between participants’ vaccination status and their characteristics. All univariable analyses were performed by Chi-square test. Significant level: 0.05.

**Table 2:** Factors associated with flu and pneumonia vaccination uptake by multivariable logistic regressions.

Characteristics (Reference)	Received a Flu Vaccination during 2020–Early 2021 Season (until Early February 2021) (Yes vs. No)	Self or Family Members Vaccinated Against Pneumonia during 2020–Early 2021 (Yes vs. No)
Female	0.86 (0.68–1.08)	0.98(0.82–1.19)
Age (≥46)		
18–25	2.32 (1.38–3.88) **	3.03 (1.99–4.61) **
26–35	1.21 (0.87–1.69)	2.04 (1.56–2.66) **
36–45	1.16(0.85–1.59)	1.31 (1.00–1.70) *
Marital status (single)		
Married	1.41 (0.96–2.09)	3.42 (2.41–4.85) **
Divorced/widow	2.06 (1.00–4.23) *	2.11 (1.02–4.39) *
Living city (Chizhou)		
Nanjing	1.33 (0.99–1.79)	0.65 (0.52–0.82) **
Shanghai	1.38 (1.00–1.90) *	0.84 (0.65–1.08)
Local registered residents	1.15 (0.85–1.54)	1.04 (0.83–1.32)
Education (Middle school or below)		
High school or technical secondary school	0.85 (0.57–1.26)	1.20 (0.90–1.59)
Junior college	0.70 (0.46–1.06)	0.82 (0.61–1.12)
Bachelor’s degree or above	0.92 (0.61–1.39)	0.92 (0.67–1.26)
Occupation (Government agency)		
Service industry	0.58 (0.44–0.77) **	0.78 (0.63–0.98) *
Manufacturing industry or agriculture	0.47 (0.31–0.72) **	0.70 (0.52–0.96) *
Others (students, unemployed et al.)	0.39 (0.27–0.57) **	0.74 (0.56–0.96) *
Annual household income (<CNY 20,000)		
CNY 20–50,000	0.80 (0.54–1.21)	0.88 (0.64–1.22)
CNY 50–100,000	0.62 (0.42–0.89) *	0.99 (0.74–1.33)
CNY 100–200,000	0.69 (0.47–1.02)	0.89 (0.65–1.23)
>CNY 200,000	0.74 (0.48–1.12)	1.07 (0.75–1.52)
Better self-rated health	1.22 (0.84–1.77)	1.49 (1.09–2.03) *
Familiarity with flu vaccine	3.02 (2.33–3.91) **	-
Perceived high susceptibility of flu	1.32 (1.00–1.75)	-
Perceived high severity of flu	1.10 (0.86–1.40)	-
Perceived high susceptibility of COVID-19	2.03 (1.42–2.89) **	1.42 (1.01–1.98) *
Perceived high severity of COVID-19	1.13 (0.85–1.49)	0.91 (0.72–1.14)
Hosmer–Lemeshow test: Chi-square	11.634	7.814
Hosmer–Lemeshow test: *p*-value	0.122	0.452

Note: Both full multivariable regression models were performed with “Enter” method and included all the covariates analyzed for each vaccine in univariable analysis. Odds ratio and 95% confidence intervals were presented. Significance level: * (*p* < 0.05); ** (*p* < 0.01).

## Data Availability

The data presented in this study are available upon reasonable request from the corresponding author.
